# An investigation of the mediating role of personality and family functioning in the association between attachment styles and eating disorder status

**DOI:** 10.1186/s40359-016-0141-4

**Published:** 2016-07-12

**Authors:** Anna Lena Münch, Christina Hunger, Jochen Schweitzer

**Affiliations:** Department of Psychology, University of Heidelberg, Hauptstraße 47-51, D-69117 Heidelberg, Germany; Institute of Medical Psychology, Center for Psychosocial Medicine, University Hospital Heidelberg, Bergheimer Straße 20, D-69115 Heidelberg, Germany

**Keywords:** Eating disorder, Attachment, Personality, Experiences in the family, Family functioning, Mediator analysis

## Abstract

**Background:**

This study examined relationships between attachment style, eating disorders (EDs), personality variables and family functioning.

**Methods:**

In our study, 253 women (*M* = 25.72 years, *SD* = 8.73) were grouped into one of four categories either according to self-reported ED diagnosis or by exceeding cut-offs for a clinical diagnosis on the *Eating Disorder Examination Questionnaire (EDE-Q)* or *Short Evaluation of Eating Disorders (SEED)*: anorexia nervosa (AN), bulimia nervosa (BN), other eating disorder (O-ED), no eating disorder (Non-ED). The ED group (AN, BN, O-ED) included 106 women (*M* = 24.74 years, *SD* = 7.71), and the Non-ED group 147 women (*M* = 26.42 years, *SD* = 9.37). Approximately half of the ED group had a comorbid disorder (59.4 %), while the majority of the Non-ED group had no psychological disorder (89.1 %).

**Results:**

Participants with an ED were significantly more often insecurely attached (*Adult Attachment Scale; AAS),* emotionally unstable, less extraverted (*Big-Five-Test of Personality; B5T)* and showed less positive family functioning (*Experiences in Personal Social Systems Questionnaire; EXIS.pers*). Results showed partial mediation for attachment and EDs through neuroticism, extraversion and family functioning.

**Discussion:**

The study found further evidence for elevated problems with attachment, personality, and family experiences in individuals with EDs, while suggesting mechanisms that may link these constructs. Implications for research and practice were discussed.

**Conclusion:**

This study supports findings that acknowledge the mediating role played by personality factors and family functioning in the relationship between attachment and EDs.

## Background

Considering eating disorders (EDs), lifetime estimated prevalence rates of anorexia nervosa (AN), bulimia nervosa (BN) and binge eating disorder were shown to be 0.40, 0.51 and 2.15 %, respectively, in an epidemiological study in Europe that also found the rates of EDs among women to be three to eight times higher than among men [42]. Since people under the age of 18 were excluded from this study, these prevalence rates should be taken as a conservative estimate of real frequencies. Numerous constructs have been found to be predictors of EDs, including genetic predispositions [6], perinatal factors [48], attachment [57], sexual and/or physical abuse [36], body image disturbance [1], dysfunctional coping strategies [50], personality disorders or accentuations [1], comorbidities [29] and poor family functioning [24].

An anxious and insecure attachment between parents and children is a consistent finding in the ED literature [57]. Attachment describes an inborn motivation to seek physical and mental proximity to a caregiver in order to find safety and care [8]. It develops between the 6^th^ and 36^th^ months of life [2], and it is transmitted cross-generationally [47]. Attachment experiences are internalized to an internal working model (IWM), representing assumptions that the child forms about itself and others [30]. Ainsworth [2] classifies secure, insecure-ambivalent, insecure-avoidant and disorganized attachment styles. The first three styles belong to organized behavior, the latter to disorganized behavior. Based on their assumption that each IWM has a positive and a negative level, Bartholomew and Horowitz [4] postulate four attachment styles that combine the two IWMs (self/other) with the two levels (positive/negative). The four attachment styles can be assigned to Ainsworth’s styles [34]. Probably because of the protective effect of secure attachment, we find higher rates of this attachment style in non-clinical versus clinical groups [38, 51]. For a secure attachment style to develop, the primary caregiver must give lasting and sensitive care [2]: if a child grows up in an environment in which it predominantly experiences anxiety, unresolved loss, rejection and physical or emotional instability, it internalizes a self-image that it is not lovable or worthy of support. This is often the case in children who develop an ED [48]. For example, perinatal factors such as obstetric difficulty, prematurity and/or birth trauma, childhood abuse and other traumatic experiences seem to play an important role in the histories of those with an ED, which may result in overprotective and over-controlling behavior in those who try to balance the perceived instability of the child’s social environment [48, 49]. Parental over-focus on eating and weight may arise as one phenomenon, potentially mediated by socio-cultural stressors such as the ‘thin ideal body size’ [22, 48, 49]. The influence of insecure attachment on maladaptive behavior patterns, such as pathological eating to regulate negative emotions, is well demonstrated [19, 21].

EDs frequently occur with comorbid personality disorders and personality accentuations, i.e. the amplification of specific aspects of the personality but without the clinical image of a personality disorder (e.g. someone with obsessive-compulsive behavior but who does not fulfill the criteria for obsessive-compulsive a personality disorder) [56]. However, findings are inconsistent. Jacobi and colleagues [28] found AN to be associated with introversion, conformity and perfectionism, while BN appeared related to impulsivity. Claes and colleagues [11] did not find specific associations for particular ED groups, suggesting that there is considerable variance in personality features among people with an ED. Eggert and colleagues [15] conducted the first study to explore mediating relationships among attachment styles, personality characteristics and EDs. They found differences between neuroticism and extraversion: neuroticism was found to be the more robust mediator, fully mediating the relationship between attachment styles and all forms of EDs, while extraversion only partially mediated this relationship.

Just as inconsistent as the findings on the relationship between personality and EDs are the results from studies investigating family functioning in the context of EDs [33]. Family functioning is defined by the interaction of family members involving physical, emotional and psychological activities and the process by which the family operates as a social system [55]. In their systematic review of the literature, Holtom-Viesel and Allan [24] found elevated difficulties in family functioning in families with a person diagnosed with an ED compared to controls.

Research suggests that family functioning may be disturbed in those with an ED in multiple ways. For example, studies have found that women with an ED report lower levels of care, increased levels of overprotection [9], and more critical comments from their parents about their body and shape [53]. While few studies have investigated emotional and verbal abuse [31, 44], one study fournd a direct correspondence between verbally-abusive fathers and/or critical mothers and EDs, independent of ED subtypes [32]. While research of this kind suggests that disturbances in family functioning are relevant to EDs, very few studies have examined how family functioning may be related to EDs. Goossens and colleagues [19] found secure attachment to be positively associated with family functioning, suggesting that family functioning may mediate the relationship between attachment and EDs.

## Aim

Secure attachment is associated with the absence of an ED, insecure attachment with the presence of an ED. Studies have investigated a broad range of ED predictors, with research suggesting that insecure attachment, the personality dimensions of neuroticism and extraversion, and difficulties in family functioning are associated with EDs. In addition to inconsistencies in this research that require further clarification, the mechanism potentially linking these constructs require investigation, with most research to date examining these constructs in isolation. As such, the aim of the present study is to provide further examination of the association between attachment, neuroticism, extraversion, family functioning and EDs, as well as examining the mediating role of personality and family functioning in the association between attachment and EDs.

**H1:** We hypothesize that an insecure attachment style will more often be seen with EDs compared to the absence thereof (Non-ED) (see [19]).

**H2:** We expect that higher levels of neuroticism and higher or lower levels of extraversion will more often be seen with EDs compared to Non-EDs [10, 39]. Due to inconsistent findings in the literature, investigations on extraversion will be explorative.

**H3:** We hypothesize that lower levels of family functioning will be seen with EDs compared to Non-EDs [59]. Due to inconsistent findings in the literature, investigations on family functioning will be explorative.

**H4:** We assume that the associations between secure/insecure attachment and EDs/Non-EDs will be mediated by neuroticism, extraversion and family functioning.

## Methods

### Participants

In total, 253 women participated in our study. On average, they were 26 years old (*M* = 25.72, *SD* = 8.73). Almost all of them were German (Table 1). Body Mass Index and age did not differ between the ED and Non-ED groups. Significantly lower educational and occupational status, and fewer marriages or partnerships, were found in the ED group. Because these variables did not correlate with the dependent variables, they were not considered in further analyses.

**Table 1:** Eating disorder pathology and demography in the total sample as well as separated for the ED and Non-ED group

	Total (*n* = 253)	ED (*n* = 106)	Non-ED (*n* = 147)
	M (SD)	M (SD)	M (SD)
Eating disorder pathology			
EDE-Q	2.29 (1.84)	3.81 (1.61)	1.19 (1.05)
SEED AN total score	0.85 (0.65)	1.33 (0.70)	0.50 (0.30)
SEED BN total score	0.75 (0.90)	1.43 (0.98)	0.25 (0.35)
Depressiveness			
PHQ	9.85 (7.36)	15.45 (7.16)	5.81 (4.18)
Body mass index (BMI)	21.27 (4.24)	20.69 (5.37)	21.68 (3.14)
Age (years)	25.72 (8.73)	24.74 (7.70)	26.42 (9.37)
Educational background^a^			
Without high school diploma	59 (23.3 %)	47 (44.3 %)	12 (8.2 %)
High school diploma	126 (49.8 %)	46 (43.4 %)	80 (54.4 %)
University degree	68 (26.9 %)	13 (12.3 %)	55 (37.4 %)
Marital status^a^			
Married/in relationship	127 (50.2 %)	41 (38.7 %)	86 (58.5 %)
Separated/single	126 (49.8 %)	65 (61.3 %)	61 (41.5 %)
Employment status^a^			
In training/studying	169 (66.8 %)	59 (55.7 %)	110 (74.8 %)
Employed	66 (26.1 %)	32 (30.2 %)	34 (23.1 %)
Not employed (unemployed, pension)	18 (7.1 %)	15 (14.2 %)	3 (2 %)
Nationality			
German	242 (95.7 %)	103 (97.2 %)	139 (94.6 %)
Other nationality	11 (4.3 %)	3 (2.8 %)	8 (5.4 %)

*ED* eating disorder group, *Non-ED* comparison group

^a^Significant between-group difference between the ED and Non-ED group regarding educational background, marital and employment status; however, none of these variables was associated with the dependent variable

The sample was divided into 106 women in the ED group (*M* = 24.74 years, *SD* = 7.71) and 147 women in the Non-ED group (*M* = 26.42 years, *SD* = 9:37). In the ED group, 86 women self-reported having an ED (AN = 45; BN = 29; other ED = 12), and 20 women exceeded the clinical cut-off in either the EDE-Q or SEED (Table 1).

### Group assignment

The categorization of participants to the ED or Non-ED group followed (1) their self-report statement (AN, BN, other kind of ED, no ED), or (2) the crossing of the clinical cut-off (>4) in the *Eating Disorder Examination-Questionnaire (EDE-Q)* or on the anorexia or bulimia subscale (>2) in the *Short Evaluation of Eating Disorders Questionnaire (SEED).*

### Comorbid disorders

With regard to comorbid disorders, 63 women (59.4 %) in the ED group had at least one: depressive disorders (14 women; 22 %); anxiety disorders (5 women; 8 %); depression and anxiety disorders (5 women; 8 %); personality disorders (3 women; 5 %); depression and personality disorders (15 women; 24 %); depression, anxiety and personality disorders (8 women; 13 %); substance abuse, schizophrenia or somatoform disorders (13 women; 20 %). The women had lived with an ED for approximately 10 years (*M* = 9.57, *SD* = 7.68). In the Non-ED group, 131 women (89.1 %) reported no comorbid disorder, while 16 women (10.9 %) reported having sought psychological treatment for depressive disorders (7 women; 4.8 %), adaption disorders (4 women; 2.7 %), somatoform disorders (1 woman; 0.7 %) or depression and personality disorders (4 women; 2.7 %).

In order to more closely examine the patients’ general psychopathology, we also assessed depressive symptoms using the depression scale in the *Patient Health Questionnaire* (PHQ-D; [20]). The ED group could be classified as a minor depressive group compared to the non-depressive Non-ED group (*t* (142.59) = 12.42, *p* < .001, *d* = 1.58). This finding is consistent with Löwe and colleagues [35], who showed that Non-ED groups exhibit some − but not clinical − depressive symptoms, whereas ED groups often demonstrate major depressive disorders (Table 1).

### Design and procedure

This cross-sectional study was conducted using an online survey based on the open-source software LimeSurvey (www.limesurvey.org). The questionnaire took about 30 min to complete. For recruiting female participants with and without an ED, we distributed flyers and study information online (e.g. via Facebook), placed public announcements in the local and online press, and we exploited our professional network with psychological psychotherapists, hospitals and psychosocial counseling centers in Germany. Participants were informed about the goals, terms and conditions of the study, as well as the requirement to be female to participate. Participants provided written informed consent before participating in our study.

### Inclusion and exclusion criteria

Inclusion criteria required participants: 1) to be assigned to either the ED or the Non-ED group; 2) to agree to participate in the study; 3) to be between 18 and 65 years of age. Exclusion criteria were lack of informed consent and/or an age out of the study range.

## Measures

### Eating disorder pathology

To examine pathological eating habits, we used the *Eating Disorder Examination Questionnaire* (EDE-Q; [16, 23]). The EDE-Q encompasses four subscales: *Restraint*, *Eating Concern*, *Weight Concern* and *Shape Concern*. The possible values between 0 and 6 indicated no ED symptoms in the lower end of the range and a distinct ED pathology in the higher end. This study used the 22 items relevant for quantitative evaluation. The EDE-Q has shown an internal consistency of *α =* .97 and has been validated with various instruments [43]. The internal consistency of the EDE-Q in the present study was α = .98.

The *Short Evaluation of Eating Disorders* (SEED; [5]) examines ED-specific behaviors for anorexia and bulimia using 11 items; the total score ranges from 0 to 3. Clinical relevance in the ED pathology can be assumed for values meeting or exceeding the cut-off (>2). The SEED showed an internal consistency for the AN subscale of *α =* .25-.40 and for the BN subscale of *α =* .32-.36. This instrument has been validated in both clinical and non-clinical samples [54]. The internal consistency of the SEED in the present study with respect to the AN subscale was *α =* .60 and with respect to the BN subscale *α =* .74*.*

### Attachment style

The attachment style was measured using the *Adult Attachment Scale* (AAS; [12, 46]). With 18 items, the AAS encompasses the subscales *Closeness*, *Dependence* and *Anxiety*. A predefined evaluation algorithm makes the assignment to categorical attachment style according to Bartholomew and Horowitz [4]. The internal consistency for the subscale *Closeness* was *α =* .51-.64, for the subscale *Dependence α =* .78 - .83, and for the subscale *Anxiety α =* .64-.76 [46]. The validity of the AAS has been confirmed repeatedly [12, 25]. The internal consistency of the AAS in the present study was *α =* .87 (*Closeness*), *α =* .90 (*Dependence*) and *α =* .91 (*Anxiety*).

The AAS and its evaluation algorithm are still in the development stage for the German version. Had we used the suggested evaluation algorithm [46] in its current form, we might have lost study participants, because they would not have been categorized clearly to one attachment style or another. After consultation with the authors of the AAS, we decided to apply a slightly modified evaluation algorithm. This procedure enabled an unambiguous classification (Table 2) that depicted the distribution of attachment styles throughout the entire sample [51].

**Table 2:** Attachment style, personality and perception of the family in the total sample and separately for the ED and Non-ED groups

	Total (*n* = 253)	ED (*n* = 106)	Non-ED (*n* = 147)
	M (SD)	M (SD)	M (SD)
Attachment style (AAS)			
Secure	112 (44.3 %)	14 (13.2 %)	98 (66.7 %)
Insecure	141 (55.7 %)	92 (86.8 %)	49 (33.3 %)
Clingy/dependent	36 (14.2 %)	12 (11.3 %)	24 (16.3 %)
Rejecting/distanced	18 (7.1 %)	7 (6.6 %)	11 (7.5 %)
Anxious/avoidant	87 (34.4 %)	73 (68.9 %)	14 (9.5 %)
Personality (B5T)			
Extraversion	26.62 (6.20 %)	23.07 (6.11 %)	29.19 (4.86 %)
Low-range values	61 (24.1 %)	52 (49.1 %)	9 (6.1 %)
Mid-range values	132 (52.2 %)	43 (40.6 %)	89 (60.5 %)
High-range values	60 (23.7 %)	11 (10.4 %)	49 (33.3 %)
Neuroticism	26.77 (7.18 %)	31.23 (6.26 %)	23.55 (5.99 %)
Low-range values	56 (22.1 %)	8 (7.5 %)	48 (32.7 %)
Mid-range values	132 (52.2 %)	45 (42.5 %)	87 (59.2 %)
High-range values	65 (25.7 %)	53 (50 %)	12 (8.2 %)
Perception of the family (EXIS.pers)	3.99 (1.37 %)	3.10 (1.26 %)	4.65 (1.03 %)

*ED* eating disorder group, *Non-ED* comparison group

Extraversion and neuroticism: low-range values = stanine scores ranging from 1 to 3; mid-range values = stanine scores ranging from 4 to 6; high-range values = stanine scores ranging from 7 to 9; EXIS.pers: theoretical range is 0 to 6

Personality was examined using the *Big*-*Five Personality Test* (B5T; [45]), which detects the same factors as the *NEO Personality Inventory* [14]. The factors *Extraversion* and *Neuroticism* were measured using 10 items each. The analysis was based on stanine values from 1 to 9 and mean values from 10 to 40. Low scores on *Extraversion* described a more withdrawn and less outgoing person, while high scores on *Extraversion* indicated unmet needs for interpersonal contact and external stimulation. Low scores on *Neuroticism* described a person who is less anxious, rarely brooding and emotionally stable, while high scores characterized an anxious and emotionally unstable person.

In the present study, the corresponding age norms were taken into account. The B5T was validated by a factor solution similar to that of Costa and McCrae [14]. Internal consistency for *Extraversion* was *α* = .87, and for *Neuroticism α = .90* [45]. The internal consistency of the subscale *Extraversion* in the present study showed *α* = .90, and that of the subscale *Neuroticism* showed *α =* .91.

### Family functioning

The *Experiences in Personal Social Systems Questionnaire* (EXIS.pers; [26, 27]) examines experiences within the family. Participants were asked to evaluate their own family experience over the last 2 weeks. The EXIS.pers includes four dimensions: *Belonging*, *Autonomy*, *Accord,* and *Confidence*. The statements are Likert items with a scale from 0 to 6; higher values indicated more positive experiences in the family system. The EXIS has been validated on 634 adults from the general population and, for the total scale, showed an internal consistency of *α* = .91. The internal consistency of the EXIS.pers in the present study was *α* = .97.

### Depression

In order to collect more information about the depressive symptoms often associated with an ED, we used the *depression* subscale of the *Patient Health Questionnaire* (PHQ-D; [35]). Based on 9 items, it was possible to achieve values between 0 and 27: values below 5 indicated no depressive symptoms, values between 5 and 10 indicated light or subliminal symptoms, and values above 10 suggested distinct depressive symptoms. The internal consistency of the *depression* subscale was *α* = .88. The PHQ-D was assessed using a validated outpatient sample [20]. The internal consistency of the PHQ-D in the present study was *α* = .93.

### Statistical analyses

The analyses were conducted using IBM SPSS Statistics 20.0. We used *χ*^2^-tests to analyze categorical data, *t*-tests for group mean differences and univariate analysis of variance for global group differences. Three exploratory mediator analyses were performed to examine the extent to which EDs (ED/Non-ED; dependent variable Y) and attachment styles (secure/insecure; independent variable X) were mediated (M) by personality (extraversion, neuroticism) and family functioning [3]. The significance of a mediator was examined on the basis of four paths: (1) the total effect of X on Y (path c), (2) the effect of X on M (path a) and (3) the effect of M on Y (path b). It was investigated whether the direct effect of X on Y differed significantly from 0, in terms of the mediator (path c’). The significance of the mediator was analyzed using Bootstrapping (*N* = 1000) to calculate the indirect effect. A mediator demonstrated significance in case of zero being outside the 95 % confidence interval. The mediator analyses were conducted using the *SPSS Syntax Indirect Macro Script* and the *Sobel Script* [40, 41]. The online survey did not permit missing data. Consequently, the statistical analyses were based on 253 full records.

## Results

**H1.** As expected, women in the ED group were significantly more likely to have an insecure attachment style (preoccupied, dismissive, fearful), and women in the Non-ED group exhibited a secure attachment style significantly more frequently (*χ*^2^ (3) = 103.99, *p* < .001, *d* = 1.67; Table 2).

**H2.** As expected, the ED group differed from the Non-ED group in their scores on neuroticism (*χ*^2^ (2) = 62.80, *p* < .001, *d* = 1.15, for stanine-values; *t* (251) = 9.86, *p* < .001, *d* = 1.26, for mean values; Table 2). In the Non-ED group, 92 % of the women described themselves as average (59 %) to below-average (33 %) in terms of emotional instability. In the ED group, 93 % of the women described themselves as average (43 %) to above-average (50 %) in terms of emotional instability. A higher ED pathology measured by the EDE-Q was associated with a higher score on neuroticism (Table 3).

**Table 3:** Eating disorder pathology and perception of the family in connection with low-,mid- and high-range values of extraversion and neuroticism

	M (SD)	M (SD)	M (SD)
ED group (*n* = 106)	Extraversion		
	Low-range values	Mid-range values	High-range values
	(*n* = 52)	(*n* = 43)	(*n* = 11)
Eating disorder pathology (EDE-Q)	4.22 (1.31)	3.67 (1.70)	2.44 (1.81)
Perception of the family (EXIS.pers)	2.54 (1.08)	3.45 (1.06)	4.24 (1.54)
	Neuroticism		
	Low-range values	Mid-range values	High-range values
	(*n* = 8)	(*n* = 45)	(*n* = 53)
Eating disorder pathology (EDE-Q)	2.60 (1.85)	3.20 (1.74)	4.52 (1.06)
Perception of the family (EXIS.pers)	4.39 (1.59)	3.47 (1.13)	2.57 (1.07)
Non-ED group (*n* = 147)	Extraversion		
	Low-range values	Mid-range values	High-range values
	(*n* = 9)	(*n* = 89)	(*n* = 49)
Eating disorder pathology (EDE-Q)	1.63 (1.42)	1.25 (1.05)	1.00 (0.96)
Perception of the family (EXIS.pers)	4.34 (1.12)	4.60 (1.07)	4.80 (0.94)
	Neuroticism		
	Low-range values	Mid-range values	High-range values
	(*n* = 48)	(*n* = 87)	(*n* = 12)
Eating disorder pathology (EDE-Q)	4.22 (1.31)	3.67 (1.70)	2.44 (1.81)
Perception of the family (EXIS.pers)	5.01 (0.98)	4.53 (0.98)	4.13 (1.19)

Extraversion and neuroticism: low-range values = stanine scores ranging from 1 to 3; mid-range values = stanine scores ranging from 4 to 6; high-range values = stanine scores ranging from 7 to 9; EDE-Q: theoretical range is 0 to 6; EXIS.pers: theoretical range is 0 to 6

As expected, the ED group differed from the Non-ED group in terms of the scores on extraversion in the exploratory examination (*χ*^2^ (2) = 65.48, *p* < .001, *d* = 1.18, for stanine values; *t* (193.54) = 8.55, *p* < .001, *d* = 1.09, for mean values; Table 2). In the Non-ED group, 94 % of the women described themselves as average (61 %) to above-average (33 %) in terms of extraversion. In the ED group, 90 % of the women described themselves as average (41 %) to below-average (49 %) in terms of extraversion. A higher ED pathology measured by the EDE-Q was associated with a lower score on extraversion (Table 3).

**H3**. As expected, the ED group disclosed less positive experiences in their families compared to the Non-ED group (*t* (197.11) = 10.52, *p* < .001; *d* = 1.34; Table 2).

**Fig. 1:**
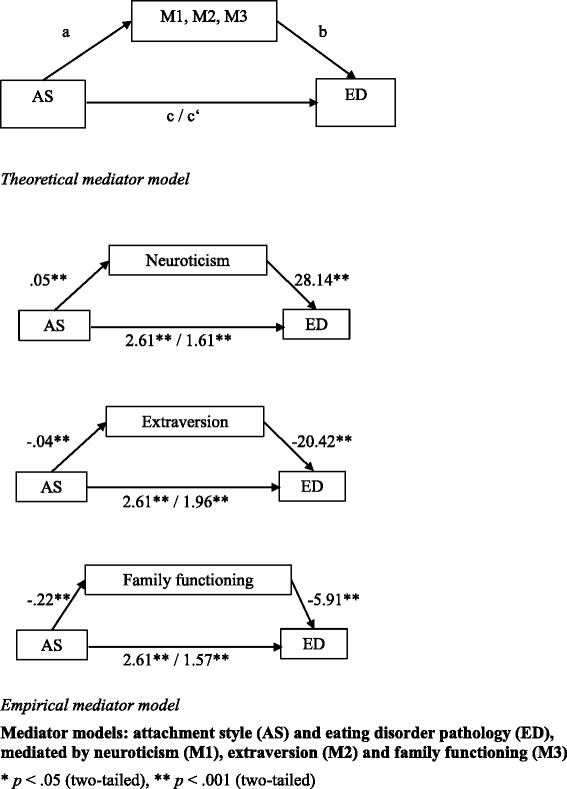
Mediator models

**H4.** We performed the following mediator analyses. The z-standardized β-coefficients of the regressions are shown in Fig. 1.

*M*_*1*_*Neuroticism.* The analysis of *neuroticism* showed that the indirect effect was significant (95 % CI [0.65 – 1.98]). Because the direct effect (*β*_*c’*_ = 2.61, *t* (251) = 7.67, *p* < .001) was not diminished in its significance by the total effect (*β*_*c*_ = 1.61, *t* (251) = 4.07, *p* < .001), a partial mediation was assumed, i.e. both insecure attachment and emotional instability appeared to be associated with an ED.

*M*_*2*_*Extraversion.* The analysis of *extraversion* revealed an indirect effect (95 % CI [0.45–1.31]). The significance of the direct effect did not change with respect to this mediator but remained highly significant (*β*_*c’*_ = 1.96, *t* (251) = 5.32, *p* < .001). A partial mediation was assumed, i.e. both insecure attachment and introversion appeared to be associated with an ED.

*M*_*3*_*Experiences in the Family*. The analysis of *experiences in the family* showed an indirect effect (95 % CI [0.79–1.92]). The direct effect was only slightly reduced by the total effect (*β*_*c’*_ = 1.57, *t* (251) = 4.04, *p* < .001) and remained highly significant, again indicating a partial mediation, i.e. both insecure attachment and less positive experiences in the family appeared to be associated with an ED.

Additional analyses revealed that women with secure attachment experienced themselves more positively within their family (*M* = 4.93, *SD* = 0.81) compared to women with insecure attachment (*M* = 3.25, *SD* = 1.26; *t* (241.16) = 12.86, *p* < .001, *d* = 1.63). It was also found that higher ED pathology, below-average scores on extraversion and above-average scores on neuroticism were associated with less positive perceptions of the family (Table 3).

## Discussion

This study examined attachment style and EDs, as mediated by personality and family functioning. As far as the authors know, this is the first study to focus on these specific constructs in a single examination with extensive sampling and by applying new instruments. The Adult Attachment Scale (AAS), the Big Five Personality Test (B5T) and the Experience in Personal Social Systems Questionnaire (EXIS.pers) are either recently published, in the process of publication, or were used for the first time on the topic of this study.

As indicated by previous work [19], this study detected a significant association between an insecure attachment style and EDs. However, due to the high inter-correlations between the insecure attachment styles [7], along with the performance of a dichotomous aggregation (secure/insecure attachment), no statements could be made about the relation of individual attachment styles to distinct ED pathologies, as was done in other studies [51]. Regarding personality accentuations, our study supports findings that demonstrate relationships between neuroticism, introversion and EDs [10, 39, 52]. Neuroticism and introversion are both associated with negative affect. Thus one possible interpretation of their relationship with EDs is that the individual engages in eating disordered behaviors in an attempt to regulate negative affect [37].

Our study also supports the assumption that an insecure attachment style was associated with negative perceptions of the family and higher ED pathology. Secure attachment was found to be associated with a more positive perception of the family and less severe ED psychopathology. Pace and colleagues [38] postulated that a secure bond to and a positive relationship with the parents act as a protective factor against EDs. The three mediator analyses showed partial mediation of attachment style and EDs by personality aspects and family functioning. Consequently, attachment styles still exert some direct effect on the ED pathology. However, personality and family functioning also accounted for variance in EDs. For future research, it would be worthwhile to investigate whether extraverted behavior and a high level of positive family functioning may have a protective influence against EDs. In contrast, the present findings indicated that emotional instability, introversion and a negative perception of the family were linked to EDs.

From a developmental perspective, our findings fit well into neurodevelopmental models of EDs that position individual and interpersonal stress as a central component in its aetiology. For AN, predispositions such as an insecure attachment style as well as emotion, stress and hypothalamic-pituitary-adrenal (HPA) axis dysregulation seem to induce a chronic stress reaction, which in turn may influence ED pathology [13]. Against the background of our findings, introversion and deficits in family functioning could be understood as involving a lack of resources to cope well with individual and social stress produced by insecure attachment, which may result in ED pathology. In future research, it would be recommended to include biopsychological parameters in a single study with personality and family variables. This would enrich our understanding of the tangled interplay of these variables in these complex disorders.

### Implications for research and practice

New instruments were applied (AAS, B5T, EXIS.pers), which appeared to be useful for future research. The evaluation algorithm for AAS [46] had been slightly modified, thus allowing the unambiguous assignment of all participants to an attachment style category, with distributions of attachment styles similar to the general population [51]. B5T and EXIS.pers both are short questionnaires that, in future studies, could be useful for the time-effective gathering of information on personality and family perceptions.

This study indicated the significance of exploring extraverted and neurotic behavior, as well as positive experiences in the family, to enhance our understanding of the complex relationship between attachment styles and eating pathology. The current finding of a relationship between an insecure attachment and EDs supports therapeutic recommendations to provide ED patients with a secure and stable therapeutic alliance in order to counteract the insecure attachment that may have developed in the family of origin. A safe (therapeutic) relationship can influence perceived and enacted emotional instability by providing functional strategies for emotion regulation. Family experiences should be addressed to uncover cognitive, behavioral and emotional (dys)functional schemas, as well as relationship experiences, so that the individual can learn healthier coping strategies and better understand the places and resources of each member in his/her important social systems. Various kinds of therapy – cognitive behavioral, psychodynamic, systemic and family therapy, as well as acceptance and commitment therapy – offer useful ways for approaching new and corrective behavioral, cognitive, emotional and relationship experiences.

### Limitations

This study was conducted online using self-reports; evaluation by others was not performed. The participants were not interviewed clinically, with the result that the diagnostic status of an ED as a primary diagnosis was not clarified completely. Future studies should focus more on clinical diagnostics (e.g. SCID-interviews; [17, 58]). Participants did not have to be in an acute state of an ED, which may have resulted in the lack of group differences with respect to the BMI. For EDE-Q there was no clinical cut-off known from German studies; consequently, we utilized the conservative cut-off resulting from US validation studies. The AAS was still in psychometric examination but seemed reliable and valid for the present study. It would be desirable to examine attachment styles using the Adult Attachment Interviews (AAI; [18, 59]) in further research. Regarding family experiences, there was no discrimination between family of origin and current family; future studies should do so explicitly. In order to gather reliable descriptions of the family of origin and the current family, the relationship status of parents and participants, as well as the family atmosphere, should be measured explicitly.

## Conclusion

The results of the present study were consistent with previous research linking insecure attachment, neuroticism, introversion, and disturbances in family functioning with EDs, as well as suggesting that the relationship between insecure attachment and EDs is partially mediated by neuroticism, introversion, and less positive family experiences. Future research of this kind examining the mechanisms through which risk factors for EDs are connected would be of benefit.

## Abbreviations

AAS, Adult Attachment Scale; AN, anorexia nervosa; B5T, Big Five Test of Personality; BN, bulimia nervosa; ED, eating disorder; EDE-Q, Eating Disorder Examination; EXIS.pers, Experiences in Personal Social Systems Questionnaire; IWM, internal working models; Non-ED, no eating disorder; O-ED, other eating disorder; PHQ-D, Patient Health Questionnaire; SEED, Short Evaluation of Eating Disorders

## References

[1] Abbate-Daga G, Gramaglia C, Amianto F, Marzola E, Fassino S (2010). Attachment insecurity, personality and body dissatisfaction in eating disorders. J Nerv Ment Dis.

[2] Ainsworth M. Patterns of Attachment. Hillsdale: Erlbaum; 1978.

[3] Baron RM, Kenny DA (1986). The moderator-mediator variable distinction in social psychological research: Conceptual, strategic, and statistical considerations. J Pers Soc Psychol.

[4] Bartholomew K, Horowitz LM (1991). Attachment styles among young adults: A test of a four-category model. J Pers Soc Psychol.

[5] Bauer S, Winn S, Schmidt U, Kordy H (2005). Construction, scoring and validation of the Short Evaluation of Eating Disorders (SEED). Eur Eat Disord Rev.

[6] Blanz B, Remschmidt H, Schmidt MH, Warnke A (2006). Psychische Störungen im Kindes- und Jugendalter [Mental disorders in childhood and adolescence].

[7] Bosmans G, Braet C, Van Vlierberghe L. Attachment and symptoms of psycho-pathology: Early maladaptives schemas as a cognitive link. Clin Psychol Psychother. 2010;17:374–85. doi:10.1002/cpp.667. 10.1002/cpp.667 20013761

[8] Bowlby J. Separation anxiety. Int J Psychoanal*.*1960a 41; 89-113. 13803480

[9] Caglar-Nazali HP, Corfield F, Cardi V, Ambwani S, Leppanen J, Olabintan O, Treasure J (2014). A systematic review and meta-analysis of ‘Systems for Social Processes’ in eating disorders. Neurosci Biobehav Rev.

[10] Cassin SE, von Ranson KM (2005). Personality and eating disorders: A decade in review. Clin Psychol Rev.

[11] Claes L, Vandereycken W, Luyten P, Soenens B, Pieters G, Vertommen H (2006). Personality prototypes in eating disorders based on the big five model. J Personal Disord.

[12] Collins N, Read S (1990). Adult attachment, working models, and relationship quality in dating couples. J Pers Soc Psychol.

[13] Connan F, Campbell IC, Katzman M, Lightman SL, Treasure J (2003). A neurodevelopmental model for anorexia nervosa. Physiol Behav.

[14] Costa PT, McCrae RR. (1992). Revised NEO Personality Inventory (NEO PI-R) and NEO Five-Factor Inventory (NEO-FFI): Psychological Assessment Resources.

[15] Eggert J, Levendosky A, Klump K (2007). Relationships among attachment styles, personality characteristics, and disordered eating. Int J Eat Disord.

[16] Fairburn CG, Beglin SJ. Assessment of eating disorders: interview or self-report questionnaire? Int J Eat Disord. 1994;16(4):363–70. doi:10.1002/1098-108X. 7866415

[17] Fydrich T, Renneberg B, Schmitz B, Wittchen H-U. SKID-II. Interviewheft [SCID-II. Interviewer manual]. Göttingen: Hogrefe; 1997.

[18] George C, Kaplan N, Main M. Adult Attachment Interview. Unpublished manuscript, University of California at Berkeley; 1985.

[19] Goossens L, Braet C, van Durme K, Decaluwé V, Bosmans G. The parent-child relationship as predictor of eating pathology and weight gain in preadolescents. J Clin Child Adolesc Psychol. 2012;41(4):445–57. doi:10.1080/15374416.2012.660690. 10.1080/15374416.2012.660690 22432541

[20] Gräfe K, Zipfel S, Herzog W, Löwe B. Screening psychischer Störungen mit dem ‘Gesundheitsfragebogen für Patienten (PHQ-D)’: Ergebnisse der deutschen Validierungsstudie. [Screening for psychiatric disorders with the Patient Health Questionnaire (PHQ). Results from the German validation study. Diagnostica]. 2004;50(4):171–81. doi:10.1026/0012-1924.50.4.171.

[21] Hertz P, Addad M, Ronel N (2012). Attachment styles and changes among women members of overeaters anonymous who have recovered from binge-eating-disorder. Health Soc Work.

[22] Hilbert A, Pike KM, Goldschmidt AB, Wilfley DE, Fairburn CG, Dohm F-A, Striegel Weissman R (2014). Risk factors across the eating disorders. Psychiatry Res.

[23] Hilbert A, Tuschen-Caffier B, Karwautz A (2007). Eating Disorder Examination - Questionnaire: Evaluation of the German translation. Diagnostica.

[24] Holtom-Viesel A, Allan S (2014). A systematic review of the literature on family functioning across all eating disorder diagnoses in comparison to control families. Clin Psychol Rev.

[25] Horowitz LM, Strauß B, Kordy H. Das Inventar zur Erfassung interpersonaler Probleme. Testmanual der deutschen Version (IIP-D) [The Inventory of Interpersonal Problems. Test manual of the German version (IIP-D)]. Weinheim: Beltz; 1994.

[26] Hunger C, Bornhäuser A, Link L, Geigges J, Voss A, Weinhold J, Schweitzer J. The Experience in Personal Social Systems Questionnaire (EXIS.pers.): Development and psychometric properties. Family Process. 2016 doi: 10.1111/famp.12205 10.1111/famp.12205 26858173

[27] Hunger C, Schweitzer J. Erleben in sozialen Systemen (EXIS) [Experience in social systems (EXIS)]. In: Kemper CJ, Brähler E, Zenger M, editors. Psychologische und sozialwissenschaftliche Kurzskalen: Standardisierte Erhebungsinstrumente für Wissenschaft und Praxis [Psychological and social-scientific short scales: Standardized assessment instruments for science and practice]. Berlin: Medizinisch Wissenschaftliche Verlagsgesellschaft; 2014. p. 76–9.

[28] Jacobi C, Paul T, Thiel A. Essstörungen [Eating disorders]. Göttingen: Hogrfe; 2005.

[29] Karwautz A, Rabe-Hesketh S, Treasure JL (2002). Pre‐morbid psychiatric morbidity, comorbidity and personality in patients with anorexia nervosa compared to their healthy sisters. Eur Eat Disord Rev.

[30] Keating L, Tasca GA, Gick M, Ritchie K, Balfour L, Bissada H (2014). Change in attachment to the therapy group generalizes to change in individual attachment among women with binge eating disorder. Psychotherapy.

[31] Kong S, Bernstein K (2009). Childhood trauma as a predictor of eating psychopathology and its mediating variables in patients with eating disorders. J Clin Nurs.

[32] Krug I, Fuller-Tyszkiewicz M, Anderluh M, Bellodi L, Bagnoli S, Collier D, Micali N (2015). A new social-family model for eating disorders: A European multicentre project using a case–control design. Appetite.

[33] Kumnig M, Höfer S, Huber A, Messner C, Renn D, Mestel R, Rumpold G. Muster dysfunktionaler Erziehungsstile und psychische Störungen im Erwachsenenalter. [ Patterns of dysfunctional parenting styles and psychological disturbances in offspring]. Z Psychosom Med Psychother. 2013;59(4):356–68. 24307335

[34] Lopez FG (1995). Contemporary Attachment Theory: An introduction with implications for counselling psychology. Couns Psychol.

[35] Löwe B, Spitzer RL, Zipfel S, Herzog W. Deutsche Übersetzung und Validierung des „Patient Health Questionnaire“(PHQ) [German translation and validation of the "Patient Health Questionnaire" (PHQ)]. Heidelberg: Medizinische Universitätsklinik; 2002.

[36] Neumark-Sztainer D, Story M, Hannan PJ, Beuhring T, Resnick MD (2000). Disordered eating among adolescents: Associations with sexual/physical abuse and other familial/psychosocial factors. Int J Eat Disord.

[37] Overton A, Selway S, Strongman K, Houston M (2005). Eating disorders. The regulation of positive as well as negative emotion experience. J Clin Psychol Med Settings.

[38] Pace U, Cacioppo M, Schimmenti A (2012). The moderating role of father’s care on the onset of binge eating symptoms among female late adolescents with insecure attachment. Child Psychiatry Hum Dev.

[39] Podar I, Jaanisk M, Allik J, Harro J. Psychological traits and platelet monoam-ine oxidase activity in eating disorder patients: Their relationship and stability. Progress in Neuro-Psychopharmacology & Biological Psychiatry, vol. 31. 2007. p. 248–53. doi:10.1016/j.pnpbp.2006.06.025. 10.1016/j.pnpbp.2006.06.025 16901600

[40] Preacher KJ, Hayes AF (2004). SPSS and SAS procedures for estimating indirect effects in simple mediation models. Behav Res Methods Instrum Comput.

[41] Preacher KJ, Hayes AF (2008). Asymptotic and resampling strategies for assessing and comparing indirect effects in multiple mediator models. Behav Res Methods.

[42] Preti A, de Girolamo G, Vilagut G, Alonso J, de Graaf R, Bruffaerts R, Morosini P (2009). The epidemiology of eating disorders in six European countries: Results of the ESEMeD-WMH project. J Psychiatr Res.

[43] Pudel V, Westenhöfer J. Fragebogen zum Essverhalten (FEV) – Handanweisung [Questionnaire about eating behavior (FEV) - manual]. Göttingen: Hogrefe; 1989.

[44] Racine SE, Wildes JE (2015). Dynamic longitudinal relations between emotion regulation difficulties and anorexia nervosa symptoms over the year following intensive treatment. J Consult Clin Psychol.

[45] Satow, L. (2012). Big-Five-Persönlichkeitstest (B5T): Test- und Skalendokumentation [Big-Five-Personality-Test (B5T): Test and scale documentation]. Downloaded from http://www.drsatow.de.

[46] Schmidt S, Strauß B, Höger D, Brähler E. Die Adult Attchament Scale (AAS) – Teststatistische Prüfung und Normierung der deutschen Version [The Adult Attachment Scale (AAS): Psychometric evaluation and normation of the German version]. Psychother Psychosom Med Psychol. 2004;54:375–82. doi:10.1055/s-2003-815000. 10.1055/s-2003-815000 15343479

[47] Scott Brown L, Wright J (2001). Attachment theory in adolescence and its relevance to developmental psychopathology. Clin Psychol Psychother.

[48] Shoebridge PJ, Gowers SG (2000). Parental high concern and adolescent-onset anorexia nervosa: A case-control study to investigate direction of causality. Br J Psychiatry.

[49] Sonneville KR, Calzo JP, Horton NJ, Haines J, Austin SB, Field AE (2012). Body satisfaction, weight gain and binge eating among overweight adolescent girls. Int J Obes.

[50] Swanson H, Power K, Collin P, Deas S, Paterson G, Grierson D, Taylor L (2010). The relationship between parental bonding, social problem solving and eating pathology in an anorexic inpatient sample. Eur Eat Disord Rev.

[51] Tasca GA, Balfour L (2014). Attachment and eating disorders: A review of current research. Int J Eat Disord.

[52] Tasca GA, Demidenko N, Krysanski V, Bissada H, Illing V, Gick M, Balfour L (2009). Personality dimensions among women with eating disorders: Toward reconceptualizing DSM. Eur Eat Disord Rev.

[53] Tetley A, Moghaddam NG, Dawson DL, Rennoldson M (2014). Parental bonding and eating disorders: A systematic review. Eat Behav.

[54] Thiel A, Paul T. Entwicklung einer deutschsprachigen Version des Eating Disorder Inventory [Development of a German-language version of the Eating Disorder Inventory (EDI)]. Zeitschrift für Differentielle und Diagnostische Psychologie. 1988;9(4):267–78.

[55] von Schlippe A, Schweitzer J. Lehrbuch der systemischen Therapie und Beratung I: Das Grundlagenwissen [Textbook of systemic therapy and counseling I: The basic knowledge]. Göttingen: V & R; 2012.

[56] Waller G, Ormonde L, Kuteyi Y (2013). Clusters of personality disorder cognitions in the eating disorders. Eur Eat Disord Rev.

[57] Ward A, Ramsay R, Treasure J (2000). Attachment research in eating disorders. Br J Med Psychol.

[58] Wittchen H-U, Wunderlich U, Gruschwitz S, Zaudig M. SKID-I. Strukturiertes Klinisches Interview für DSM-IV. Achse I [Structured Clinical Interview for DSM-IV. Axis ]. Göttingen: Hogrefe; 1997.

[59] Zachrisson HD, Skaderud F (2010). Feelings of insecurity: Review of attachment and eating disorders. Eur Eat Disord Rev.

